# Epidermal Growth Factor Receptor Kinase Inhibitor Ameliorates β-Amyloid Oligomer-Induced Alzheimer Disease in Swiss Albino Mice

**DOI:** 10.3390/molecules27165182

**Published:** 2022-08-14

**Authors:** Jagadeesh Dhamodharan, Ganthimathy Sekhar, Arunachalam Muthuraman

**Affiliations:** 1 Unit of Anatomy, Faculty of Medicine, AIMST University, Semeling, Bedong 08100, Kedah, Malaysia; 2 Department of Pathology, Faculty of Medicine, Saveetha Institute of Medical & Technical Sciences (SIMATS), Saveetha University, Chennai 602105, Tamilnadu, India; 3 Unit of Pharmacology, Faculty of Pharmacy, AIMST University, Bedong 08100, Kedah, Malaysia

**Keywords:** acetylcholinesterase, donepezil, gefitinib, intracerebroventricular, Morris water maze, neurodegeneration

## Abstract

Alzheimer’s disease (AD) is one of the major neurodegenerative disorders, and its incidence increases globally every year. Currently, available AD drugs symptomatically treat AD with multiple adverse effects. Gefitinib (GE) is an epidermal growth factor receptor (EGFR) kinase inhibitor. EGFR is the preferred target for the treatment of AD, whereas the effect of GE in AD conditions is limited. The present study was designed to explore the ameliorative potential of GE in Aβ_1–42_ oligomer-induced neurotoxicity in AD mice. AD was induced by intracerebroventricular (i.c.v.) injection of Aβ_1–42_ oligomer (4 μg/4 μL) into the lateral ventricles of the mouse brain. The test compound, i.e., GE (2 and 4 mg/kg of body weight), was administered orally on days 10, 13, 16, 19, 22, 25, and 28, and the reference drug, i.e., donepezil (DP, 2 mg/kg), was administered orally from the 10th to 28th days. The behavioral changes were screened by the Morris water maze (MWM) test. Furthermore, biomarkers i.e., brain acetylcholinesterase (AChE), thiobarbituric acid reactive substances (TBARS), and reduced glutathione (GSH) levels were estimated from brain samples. The AD-associated histopathological changes were analyzed by hematoxylin and eosin staining. The administration of GE significantly ameliorated the AD-associated behavioral, biochemical, and histopathological changes. The ameliorative effect of GE against the Aβ_1–42_ oligomer-associated neurotoxicity was due to its potent inhibition of EGFR kinase activation, as well as its antioxidant and antilipid peroxidative effect.

## 1. Introduction

Alzheimer’s disease (AD) is one of the major neurodegenerative disorders accounting for more than 60–80% of dementia cases globally [1]. On the basis of the various hypotheses behind AD progression, researchers have proposed multiple pharmacological treatment approaches. Clinically, approved drugs for the management of AD are limited. Cholinesterase inhibitors (donepezil (DP), rivastigmine, and galantamine), glutamate regulators (memantine), orexin receptor antagonists (suvorexant), and anti-amyloid antibody molecules (aducanumab) are recommended for the management of AD [2,3]. However, these drugs also produce a variable degree of side-effects such as loss of appetite, increased frequency of bowel movements, constipation, mental confusion, and dizziness [4,5,6]. Moreover, various targets have been explored in AD management, whereas selective target active agents and test reports in experimental animals are limited.

In AD conditions, the nervous system undergoes progressive neuron cell loss and decreases the dendritic neuron sprouting process, which leads to enhanced brain shrinkage, i.e., atrophy, followed by neuronal death [7]. The primary symptoms of AD are declining thinking ability, alternated neuronal behavioral and social skills, and effects on the day-to-day lifestyle [2,8]. The regulation of dendritic neuron connection with the neuronal sprouting process can ameliorate AD and other neurodegenerative disorders [9]. Furthermore, the accumulation of amyloid precursor proteins (APPs), tau (τ) proteins, and amyloid-β (Aβ) peptides are primary markers of the early stage of AD progression [10]. Experimentally, the amyloid-β oligomer is known to activate the epidermal growth factor receptor (EGFR) and also alters the intracellular kinase signaling cascade pathways which lead to effects on the neuronal sprouting process and neuronal cell growth [11]. Hence, the amyloid-β oligomer-associated alteration of EGFRs and their kinase contributes to the AD progression and can reduce the long-term potentiation of neuronal cells [9,12]. Currently, inhibition of EGFR kinase acts as a key target for the management of AD [13].

Clinically, selective inhibitors of EGFR kinase are commonly used in cancer treatments such as erlotinib, lapatinib, and gefitinib (GE) [14]. Gefitinib (GE) was the first selective inhibitor of epidermal growth factor receptor (EGFR) tyrosine kinase activation, and it regulates cell growth and cell division via interactions of antiapoptotic and Ras signaling cascade mechanisms of cells [15,16]. GE is commonly used for lung cancer, certain breast cancers, and other cancers. Initially, it was FDA-approved for non-small-cell lung cancer (NSCLC) [15,17,18]. However, it is also recommended following the failure of platinum-based compound and docetaxel chemotherapies for lung cancer patients [19]. Furthermore, epidermal growth factor receptor inhibitors can regulate amyloid-β biosynthesis and clearance in brain tissue. EGFR is one of the potential drug targets for the management of memory dysfunction [13]. Hence, newer anticancer agents such as GE can be repurposed for the management of AD via selective inhibition of EGFR actions [20]. However, in June 2005, FDA withdrew GE for usage in new cancer patients due to its lack of evidence and questionable life extension [21]. GE is now used for certain cancers, and it has also been investigated for its potential use in neuroprotection and prevention of neurodegenerative disorders due to its proposed neuronal sprouting action [20]. Hence, this study investigated the effect of an EGFR kinase inhibitor, i.e., GE, in ameliorating β-amyloid oligomer-induced AD in Swiss albino mice.

## 2. Results

### 2.1. Effect of GE on AD-Induced Behavioral Changes 

In the present study, the *i.c.v.* injection of Aβ_1–42_ oligomer into the lateral ventricle of mouse brain showed significant (*p* < 0.05) impairment of memory and cognition function in MWM tests when compared to the normal animal group. The administration of GE (2 and 4 mg/kg) significantly ameliorated the above neurobehavioral changes when compared to the AD group. The effect of GE (4 mg/kg.) showed a similar effect to that of the reference drug, i.e., DP (2 mg/kg; *p.o.*)-treated group. The details are described in the next section.

### 2.2. Effect of GE on Escape Latency Time (ELT) and Time Spent in the Target Quadrant (TSTQ) in MWM Test

**Figure 1:**
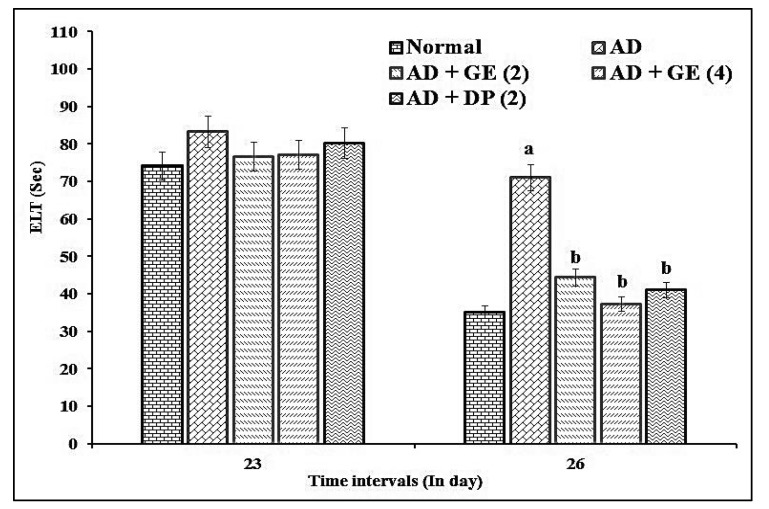
Effect of GE on ELT in MWM test assessment. Digits in parentheses indicate the dose in mg/kg. Data are expressed as the mean ± SD, *n* = 8 mice per group. ^a^ *p* < 0.05 vs. normal group; ^b^ *p* < 0.05 vs. AD group. Abbreviations: AD, Alzheimer’s disease; DP, donepezil; ELT, escape latency time; GE, gefitinib; Sec, seconds.

**Figure 2:**
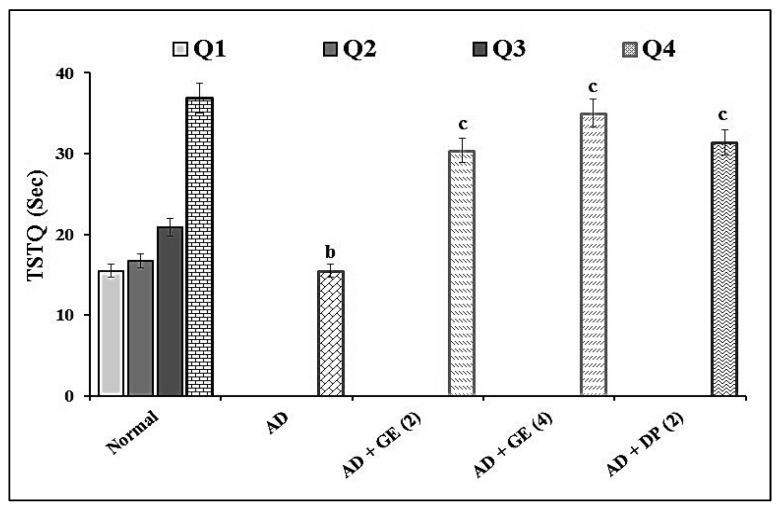
Effect of GE on TSTQ in MWM test assessment. Digits in parentheses indicate the dose in mg/kg. Data are expressed as the mean ± SD, *n* = 8 mice per group. ^b^ *p* < 0.05 vs. Q_4_ normal group; ^c^ *p* < 0.05 vs. AD group. Abbreviations: AD, Alzheimer’s disease; DP, donepezil; GE, gefitinib; Q, quadrant; Sec, seconds; TSTQ, time spent in the target quadrant.

Aβ_1–42_-induced AD mice (4 µg/4 μL; *i.c.v.*) showed significant (*p* < 0.05) impairment of cognitive dysfunction in the MWM test as an indication of increased ELT and decreased TSTQ values when compared to the normal control group. The administration of GE (2 and 4 mg/kg; *p.o.*) significantly ameliorated the above ELT and TSTQ responses when compared to the AD group. These ameliorative effects were similar to those of the reference drug, i.e., DP (2 mg/kg; *p.o.*)-treated group. The results are illustrated in Figure 1 and Figure 2.

### 2.3. Biomarker Estimations

On the 28th day, all the animals were anesthetized with diethyl ether and then sacrificed before collecting the brain tissues. Thereafter, under a stereo microscope, different parts of the brain such as the cortex, hippocampus, and cerebellum were precisely dissected for the estimation of tissue biomarker changes such as AChE, TBARS, and GSH levels. The results of all biomarker estimations are explained below.

#### Effect of GE on Aβ Oligomer-Induced AD Tissue Biomarker Changes

The administration of Aβ_1–42_ oligomer (4 µg/4 μL; *i.c.v.*) showed significant (*p* < 0.05) alteration of brain tissue biomarkers, i.e., an increase in AChE and TBARS activity and a decrease in GSH activity, in all the brain regions such as the hippocampus, cerebral cortex, and cerebellum when compared to the normal control group. The administration of GE (2 and 4 mg/kg; *p.o.*) significantly attenuated the Aβ_1–42_ oligomer-induced changes in tissue biomarkers when compared to the AD group in a dose-dependent manner. These ameliorative effects of GE were similar to the reference drug, i.e., DP (2 mg/kg; *p.o.*)-treated group. The results are indicated in Table 1, Table 2 and Table 3**.**

### 2.4. Effect of GE on AD-Induced Histopathological Changes

**Figure 3:**
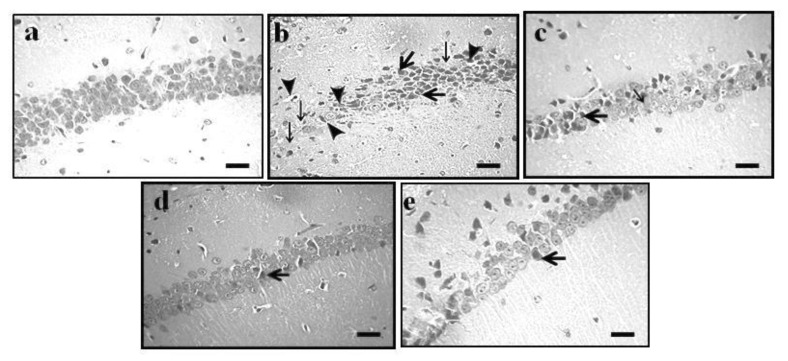
Effect of GE on AD-induced histopathological changes in CA1 region of the hippocampus. (**a**–**e**) Histopathological images of coronal sections of mouse brain tissue of normal, AD, and GE (2 and 4 mg/kg)- and DP (2 mg/kg)-treated groups, respectively. (**a**) Normal arrangement of pyramidal cells of the CA1 region and vesicular nuclei in hippocampus regions. The thin arrow shows neuron loss and neurofibrillary degeneration and neuronophagia, the thick arrow shows nuclear pyknosis, and the arrowhead shows vacuolation in the CA1 region of the hippocampus. (**b**) Typical colliquative necrosis, characterized by disrupted cell membranes, fragmented nuclei, and inflammatory cells, indicating that the Aβ oligomer caused neuronal structural pathological injuries in mice. (**c**–**e**) Reversal of Aβ_1–42_-induced histopathological changes. Microscopic examinations were performed under 400× light microscopy; scale bar = 35 µm.

**Figure 4:**
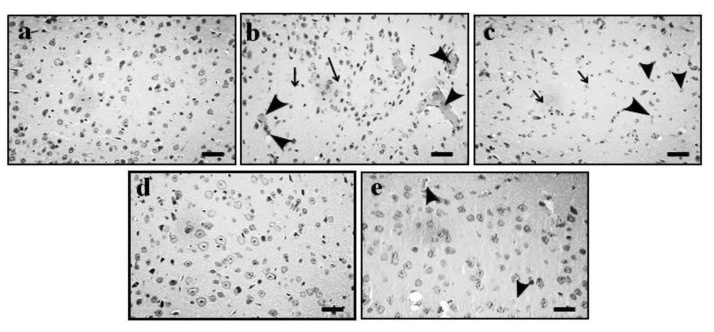
Effect of GE on AD-induced histopathological changes in the cortex region of the mouse brain. (**a**–**e**) Histopathological images of coronal sections of mouse brain tissue of normal, AD, and GE (2 and 4 mg/kg)- and DP (2 mg/kg)-treated groups, respectively. (**a**) Normal arrangement in cortex regions of the mouse brain. The thin arrow shows neuron loss and neurofibrillary degeneration and neuronophagia, the thick arrow shows nuclear pyknosis, and the arrowhead shows vacuolation in cortex regions. (**b**) Typical colliquative necrosis, characterized by disrupted cell membranes, fragmented nuclei, and inflammatory cells, indicating that the Aβ oligomer causes neuronal structural pathological injuries in mice. (**c**–**e**) Reversal of Aβ_1–42_-oligomer induced histopathological changes. Microscopic examinations were performed under 400× light microscopy; scale bar = 35 µm.

The administration of Aβ_1–42_ oligomer (4 μg/4 μL; *i.c.v.*) was shown to potentially induce AD. AD-associated neuronal changes were observed in the pyramidal cells of the Cornu Ammonis (CA1) region and vesicular nuclei in the hippocampus and cortex of mouse brain when compared to the normal animal group. The administration of GE (2 and 4 mg/kg; *p.o.*) showed significant ameliorative effects against the Aβ_1–42_-induced AD-associated histopathological changes. Furthermore, these results were similar to the reference drug (DP; 2 mg/kg) treatment. The results of the effect of GE on AD-associated histopathological changes are illustrated in Figure 3a–e (hippocampus) and Figure 4a–e (cortex).

## 3. Discussion

The administration of Aβ_1–42_ oligomer (4 µg/4 μL; *i.c.v.*) showed significant (*p* < 0.05) induction of AD, reflected by brain histopathological changes in hippocampus and cortex region such as neuron loss, neurofibrillary degeneration, neuronophagia, nuclear pyknosis, vacuolation, amyloid deposition, and tau aggregation. In the Morris water maze test, ELT was significantly increased and TSTQ was significantly decreased in the AD group, indicating a clear loss of memory in the AD group. Similarly, there was an alteration in the tissue biomarkers i.e., an increase in AChE and TBARS activity and a decrease in GSH activity in the hippocampus, cerebral cortex, and cerebellum of mouse brain, highlighting the increased oxidative stress in the AD group. However, the administration of GE (2 and 4 mg/kg; *p.o.*) and DP (2 mg/kg; *p.o.*) significantly ameliorated the Aβ_1–42_-oligomer-associated AD changes and restored the histopathological, behavioral, and tissue biomarker changes close to the normal level. These indicated a potential ameliorative effect of GE against the Aβ_1–42_ oligomer-associated AD. 

Various experimental reports evidenced that the Aβ_1–42_ oligomer enhances β-amyloid and tau protein accumulation [22]. Moreover, β-amyloid also enhances senile plaque formation and neuronal degeneration, leading to the progression of mild to severe AD [23,24]. Furthermore, the accumulation of Aβ_1–42_ tends to produce neurotoxic effects, such as inducing oxidant stress and promoting microglial activation [25,26]. Current studies also revealed that the administration of Aβ_1–42_ oligomer is known to produce free radicals (decreased GSH) and neuroinflammation (elevation of TBARS level). However, the administration of GE attenuates the Aβ_1–42_ oligomer-induced oxidative stress and neuroinflammation. Current results agree with other studies, where GE showed potential antioxidant effects via free-radical (DPPH (2,2-diphenyl l-1-picrylhydrazyl) and hydroxyl radical) scavenging actions [27,28].

Furthermore, the molecular mechanisms underlying the pharmacological and genetic effects of EGFR revealed that Aβ enhances the EGFR kinase activation in the hippocampus and cortex regions, and it can cause neuroinflammation, neurodegeneration, and memory dysfunctions [28,29]. These results also agree with other studies, where EGFR activation with Aβ_1–42_ oligomer treatment caused neurodegeneration via the expression of kinase proteins involved in EGFR/Ras signaling pathways [13]. Furthermore, a lower GE oral dose (1–5 mg/kg) with intermittent administration was reported to produce a better effect on neuroprotection than the daily dose (40 mg/kg; cancer treatment dose) regimen [13,30]. Current results and literature reports support our histopathological observations.

Furthermore, the administration of Aβ_1–42_ oligomer enhances the brain’s AChE activity (hippocampus, cortex, and cerebellum regions). This is the hallmark of Aβ_1–42_ oligomer-induced neurodegeneration and memory dysfunction [31]. The inhibitor of acetylcholinesterase activity, i.e., donepezil, is known to produce neuroprotective, antioxidant, and anti-inflammatory actions in brain tissue [32]. Similarly, GE showed a reduction in AChE against the Aβ_1–42_ oligomer-associated neurotoxicity. GE possesses potential antioxidant and anti-inflammatory properties for neuroprotective actions [27,33]. Moreover, GE is known to possess an ameliorative effect on nicotinic acid-based parasympathomimetic stimulation and arecoline-induced activation of EGFR via muscarinic acetylcholine receptor (mAChR3) [34]. However, autocrine acetylcholine readily activates EGFR signaling via muscarinic receptor-3 (M3), which leads to altering the neuronal rejuvenation process [35,36]. Hence, the treatment of GE and DP was shown to produce a reduction in AChE activity level, which is essential for the improvement of memory functions. This is the first report to reveal that the treatment of GE produced a reduction in AChE activity level and improvement of memory function against the Aβ_1–42_ oligomer toxicity.

## 4. Materials and Methods

### 4.1. Animals

In this research work, disease-free Swiss albino male mice (12 months old; 20–35 g) were used. Animals were maintained in the central animal house at AIMST University with a standard laboratory diet (Soon Soon Oilmills Sdn Bhd, Penang, Malaysia). The animal was allowed access to water ad libitum. Natural light and dark cycles (12 h/12 h) were maintained. The macro-environmental temperature and humidity of animal houses were 25 °C and 50%. The experimental protocol was approved by the AIMST University Animal Ethics Committee (AUAEC/FOM 2020/02—Amendment No. 1). Animals were cared for as per the guidelines of AUAEC.

### 4.2. Chemicals

Amyloid (Aβ_1–42_; Biotek Abadi, Cayman Chemicals, USA), gefitinib (Merck Sdn. Bhd., Selangor, Malaysia), donepezil (Alkem Laboratories Limited, Lower Parel, Mumbai, India), ketamine hydrochloride injection (Dechra Pharmaceuticals PLC, Northwich, United Kingdom), xylazine (XYLAMAX^®^, Bimeda Canada), 5,5′-dithiobis-(2-nitrobenzoic acid), acetylthiocholine iodide, thiobarbituric acid, 1,1,3,3-tetra methoxy propane, reduced glutathione, Folin-Ciocâlteu reagent, and bovine serum albumin were purchased from Merck & Co., Inc., Osaka, Japan.

### 4.3. Preparation of Aβ_1–42_ Oligomer

Aβ_1–42_ oligomer solution was freshly prepared before intracerebroventricular (*i.c.v.*) injection. Briefly, Aβ_1–42_ protein was dissolved in filtered phosphate-buffered saline (PBS: 1 μg/μL), consisting of 10 mM sodium-dihydrogen phosphate (NaH_2_PO_4_), disodium hydrogen phosphate (Na_2_HPO_4_), and 100 mM of sodium chloride (NaCl) dissolved in glass-distilled deionized water (pH = 7.5). The Aβ_1–42_ solution was then incubated at 37 °C for over 3 days before use [37].

### 4.4. Induction of AD Mouse Model

In mice, AD was induced by intracerebroventricular (*i.c.v.*) injection of Aβ_1–42_ oligomer under anesthetic condition by a mixture of ketamine (75 mg/kg) and xylazine (5 mg/kg). According to the established procedure by Paxinos and Franklin [38], a total of 4 μg/4 μL of Aβ_1–42_ oligomer solution was injected into the lateral ventricles of mouse brain (2 μL on each side) at stereotaxic coordinates (anteroposterior—0.2 mm; mediolateral—1.0 mm; dorsoventral—2.5 mm) taken from the atlas of the mouse brain [39]. Animals in the normal group received 0.9% NaCl injections, and animals in all the other groups were injected with Aβ_1–42_ solution.

### 4.5. Experimental Protocol

Five groups of male adult Swiss albino mice (*n* = 8) were employed in this study. Group I served as a normal control group. Group II served as the AD group. AD was induced by intracerebroventricular (*i.c.v.*) injection of Aβ_1–42_ (4 μg/4 μL) into the lateral ventricles of mouse brain (2 μL bilaterally). Groups III and IV served as the test compound treatment groups, i.e., gefitinib (GE) at doses of 2 and 4 mg/kg, with oral administration on days 10, 13, 16, 19, 22, 25, and 28. Group V served as the reference drug treatment group, i.e., donepezil (DP, 2 mg/kg), with oral administration from the 10th to 28th days. 

**Figure 5:**
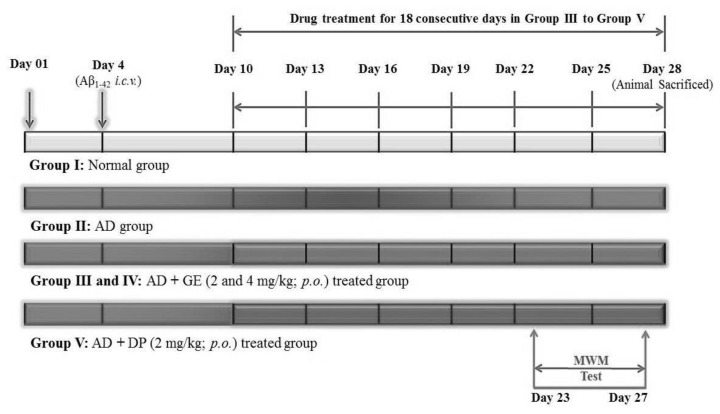
Experimental protocol for the screening of GE effect in the AD model. Abbreviations: Aβ_1–42_, beta-amyloid peptide; AD, Alzheimer’s disease; DP, donepezil; GE, gefitinib; *i.c.v.*; intracerebroventricular; MWM, Morris water maze test.

Behavioral training for the Morris water maze test (MWM) was performed from the 23rd to the 26th day, and the behavioral response was recorded on the 27th day. On the 28th day, the animal was sacrificed, and brain tissue samples were collected for histopathological evaluation and biomarker estimation. The molecular mechanisms were confirmed by estimation of biomarkers such as brain acetylcholinesterase (AChE), reduced glutathione (GSH), and thiobarbituric acid reactive substances (TBARS) activities in the hippocampus, cortex, and cerebellum of mouse brain. The micro-anatomical changes were analyzed by histopathological observation of mouse brains using hematoxylin and Eosin (H&E) stain. The details of the experimental protocol are illustrated in Figure 5.

### 4.6. Screening of the Effect of GE in AD-Associated Neurobehavioral Changes

Neurobehavioral changes associated with AD were assessed at different time intervals. Behavioral training for the Morris water maze test (MWM) was performed from the 23rd to 26th day, and, on the 27th day, the behavioral response was recorded.

#### Assessment of Learning and Memory Using the Morris Water Maze (MWM) Test

**Figure 6:**
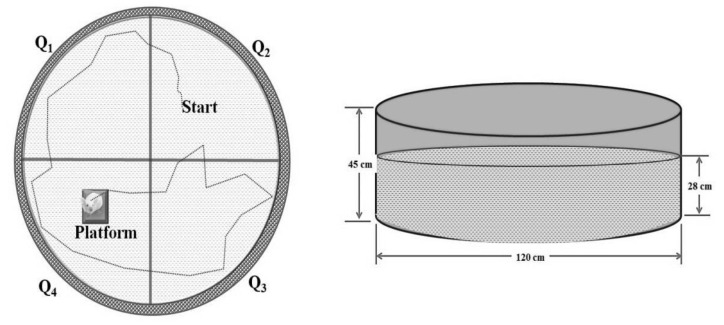
Image of MWM test apparatus. Abbreviation: Q, quadrant.

The MWM test was modified from a previously described method by Morris [40] and Parle [41]. The MWM test was conducted to assess the learning and memory of the animals. Behavioral training for MWM was performed from the 23rd to the 26th day, and the behavioral response was recorded on the 27th day. This is a swimming-based model where the animal learns to escape onto a hidden platform. The apparatus consisted of a large circular pool (150 cm in diameter, 45 cm in height, filled to a depth of 30 cm with water maintained at 37 °C ± 1 °C). The MWM test apparatus was divided with two threads fixed at right angles to each other on the rim of the pool, and the tank was divided into four equal quadrants. A submerged platform (10 cm²) was placed inside the target quadrant, 1 cm below the surface of the water (Figure 6). The position of the platform was kept unaltered throughout the training session. Each day, four consecutive training trials were given to each animal with intertrial gaps of 5 min. The mouse was gently placed in the water between quadrants, facing the wall of the pool with the drop location changing for each trial, and allowed 90 s to locate the submerged platform. The animal was allowed to stay on the platform for 20 s. If it failed to find the platform within 90 s, it was guided gently onto the platform and allowed to remain there for 20 s. Each animal was subjected to four training trials each day for four consecutive days (Table 4). The starting position was changed with each exposure as mentioned below, and the target quadrant (Q_4_) was kept constant throughout the training period. On day 4, the escape latency time (ELT) to locate the hidden platform in MWM was noted as an index of acquisition or learning.

On the fifth day, the water was made opaque with white-colored nontoxic dye. The platform was removed, and each mouse was allowed to explore the pool for 90 s. In all four quadrants, the mean time spent was noted. The mean time spent by the mouse in the target quadrant searching for the hidden platform was noted as an index of retrieval or memory. The experimenter, after dropping the animal at the pool, always stood in the same position. Proper care was taken regarding the relative location of the water maze concerning other objects in the laboratory so that prominent visual clues were not disturbed during the total duration of the study. All of the trials were completed between 9:00 a.m. and 6:00 p.m.

### 4.7. Estimations of Tissue Biomarker Changes

On the 28th day, all the animals were anesthetized with diethyl ether. Thereafter, animals were sacrificed, and brain tissues were collected for the estimation of brain biomarker changes such as AChE, TBARS, and GSH levels. The details of all biomarker estimations are explained below.

#### 4.7.1. Estimation of AChE as an Indication of Cholinergic Neurochemical Alteration in CNS

AChE levels of brain tissues were estimated using the method described by Ellman et al. [42]. Briefly, 500 μL of brain supernatant was mixed with 0.25 mL of Ellman’s reagent 5,5′-dithiobis-(2-nitrobenzoic acid) (DTNB (0.001 M)), and it was allowed to develop a yellow color chromogen. The principles of in vitro AChE estimation are described below.

$$\mathrm{Acetyl}\;\mathrm{thiocholine}\;\mathrm{iodide}\frac{\mathrm{Cholinesterase}}{}\to \mathrm{Thiocholine}+\mathrm{Acetate}\ldots \;\mathrm{Step}\;1.\;\mathrm{Thiocholine}+\mathrm{DTNB}\to \;\mathrm{Trinitrobenzoate}\;\mathrm{chromogen}\ldots \;\mathrm{Step}\;2.$$

In step 1, acetylcholine esterase reacts with acetylthiocholine iodide and forms the thiocholine and acetate products. In step 2, thiocholine reacts with DTNB to form the trinitrobenzene chromogen (yellow color). Corresponding to changes in absorbance (OD, optical density), the changes in yellow color chromogens were quantified using a spectrophotometer (DU 640B Spectrophotometer, Beckman Coulter Inc., California, USA) at 420 nm. The OD values were used for further calculation of the AChE activity level according to the following formula:

$$R=\frac{\delta \;O.D.\times \mathrm{Volume}\;\mathrm{of}\;\mathrm{the}\;\mathrm{assay}\;\left(3\;\mathrm{mL}\right)}{\varepsilon \times \mathrm{mg}\;\mathrm{of}\;\mathrm{protein}},$$

where ‘R’ is the rate of enzyme activity in ‘n’ moles of acetylthiocholine iodide hydrolyzed/min/mg of protein, ‘δ O.D.’ is the change in absorbance/min, and ‘ε’ is the extinction coefficient, i.e., 13,600/M/cm.

#### 4.7.2. Estimation of TBARS as an Indication of Lipid Peroxidation

The TBARS level of brain tissue was estimated as described by Ohkawa et al. [43]. Briefly, 0.2 mL of brain tissue supernatant of homogenate was mixed with 0.2 mL of 8.1% sodium, 0.2 mL of 8.1% sodium dodecyl sulfate, 1.5 mL of 30% acetic acid, and 1.5 mL of 0.8% of thiobarbituric acid (TBA) in a test tube. The total volume of 4 mL was made up with distilled water. Next, test tubes were incubated at 90 °C for 1 h. After that, 1 mL of distilled water was added, before centrifuging at 4000 revolutions per minute (rpm) for 10 min. A pink color chromogen was developed. The principle of in vitro TBARS estimation is described below.

$$\mathrm{MDA}+\mathrm{TBA}\to \mathrm{MDA}-{\mathrm{TBA}}_{2}.$$

In the lipid peroxidation process, the active lipid peroxidation byproducts, i.e., malondialdehyde (MDA) readily react with TBA and form the intermediate MDA–TBA_2_ adduct and water. This adduct has a pink color. Using a spectrophotometer (DU 640B Spectrophotometer, Beckman Coulter Inc., CA, USA) at 532 nm, the changes in absorbance of the pink color chromogen were recorded. A standard curve was prepared with absorbance value (y-axis) versus reference standard concentration (x-axis), i.e., 1,1,3,3-tetra methoxy propane (TMP) 0–100 nmol/mL. Using the following formula, the level of TBARS was quantified:

$$\mathrm{TBARS}\left(\frac{\mathrm{nmol}}{\mathrm{ml}}\right)=\frac{\delta \;O.D.\;\mathrm{sample}}{\varepsilon \times \mathrm{PL}\;}\times \mathrm{DF},$$

where ‘δ O.D.’ is the change in absorbance/min, ε is the extinction coefficient, i.e., 1.56 × 10^5^/m/cm, DF is the dilution factor, and PL is the path length. The calculated level of MDA (nmol/mL) value was further integrated with mg of protein. The net value of MDA was expressed as nmol of MDA per mg of protein.

#### 4.7.3. Estimation of GSH as an Indication of Oxidative Stress

The GSH level of brain tissue was estimated using the method described by Ellman [44]. Briefly, the brain tissue supernatant was mixed with 10% *w*/*v* trichloroacetic acid (1:1 ratio) to make the protein precipitations. After that, at 4 °C, the test tube samples were centrifuged at 1000 rpm for 10 min. About 0.5 mL of clear aliquot was mixed with 2 mL of 0.3 M disodium hydrogen phosphate. Thereafter, 0.25 mL of 0.001 M freshly prepared 5,5′-dithiobis (2-nitrobenzoic acid) (DTNB) solution was added. In 1% *w*/*v* sodium citrate solution, the DTNB was dissolved, and it developed a yellow color chromogen. The principle of in vitro GSH estimation is described below.

DTNB+GSH→TNB.

GSH readily reacts with DTNB and produces the yellow color chromogen product i.e., 5-mercapto-2-nitrobenzoic acid (TNB). Using a spectrophotometer (DU 640B Spectrophotometer, Beckman Coulter Inc., California, USA) at 412 nm, the changes in absorbance of yellow color chromogen were recorded. The standard curve was prepared with absorbance value (y-axis) versus reference standard concentration (x-axis), i.e., GSH 10–100 µmol/mL. The GSH level was quantified using the following formula:

$$\mathrm{GSH}=\frac{\delta \;O.D.\;\mathrm{Standard}-\left(y-\mathrm{intercept}\right)}{\mathrm{Slope}\;}\times \mathrm{DF}\times 2,$$

where ‘δ O.D.’ is the change in absorbance of the standard, y-intercept is the y-intercept of the linear curve value from the standard plot, slope is the value obtained from the standard plot, DF is the dilution factor, and 2 denotes the conversion of one oxidized glutathione to two reduced glutathione. The calculated level of GSH (µmol/mL) value was further integrated with mg of protein. The net value of GSH was expressed as µmol of GSH per mg of protein.

### 4.8. Analysis of Mouse Brain Histopathological Changes

After the completion of behavioral studies, the animals were sacrificed on the 28th day, and the mouse brains were collected. The brain tissues were fixed with neutral buffered 10% paraformaldehyde solution. The tissue block was prepared with paraffin wax. The embedded mouse brain blocks were used for the coronal sectioning under the semi-automatic cryo-microtome device. The 5 μm sectioned tissues were used for the further process of H&E staining. The stained slides were observed for histopathological changes in the hippocampus and cortex region of mice brains under 400× magnification. The histopathological changes were noted and compared with normal and reference control animal tissue sections.

### 4.9. Statistical Analysis

All results were expressed as the mean ± standard deviation (SD). The behavioral data were statistically analyzed using two-way analysis of variance (ANOVA) followed by the Bonferroni post hoc test, and the data of tissue biomarkers, i.e., AChE, TBARS, and GSH levels, were analyzed using one-way ANOVA followed by Tukey’s multiple range test using Graph pad prism version 5.0 software. A *p*-value ˂ 0.05 was considered to be statistically significant.

## 5. Conclusions

Our study showed that the administration of GE could ameliorate the Aβ_1–42_ oligomer-induced toxicity due to its potential epidermal growth factor receptor kinase-inhibitory action. Furthermore, GE also possessed free-radical scavenging action, prevented lipid peroxidation, and regulated acetylcholine levels in various parts of the mouse brain. Therefore, GE can be a novel agent for the prevention of AD and other neurodegenerative disorders such as multiple sclerosis, Parkinson’s disease, neuropathic pain, and dementia.

## Figures and Tables

**Table 1:** Effect of GE on the changes in AChE levels in brain tissue biomarkers.

Groups	Hippocampus	Cortex	Cerebellum
Normal	21.5 ± 1.5	20.7 ± 2.3	20.2 ± 1.3
AD	41.1 ± 1.3 ^a^	42.5 ± 1.2 ^a^	36.4 ± 1.5 ^a^
AD + GE (2)	28.9 ± 0.7 ^b^	29.2 ± 1.7 ^b^	30.3 ± 1.7 ^b^
AD + GE (4)	23.5 ± 1.1 ^b^	21.4 ± 1.6 ^b^	20.5 ± 1.6 ^b^
AD + DP (1)	22.3 ± 1.3 ^b^	22.9 ± 1.4 ^b^	21.3 ± 1.4 ^b^

Digits in parentheses indicate the dose in mg/kg; the value of AChE level is expressed as μmol/mg of protein/min. Data are expressed as the mean ± SD, *n* = 8 mice per group. ^a^ *p* < 0.05 vs. normal group; ^b^ *p* < 0.05 vs. AD group. Abbreviations: AD, Alzheimer’s disease; DP, donepezil; GE, gefitinib.

**Table 2:** Effect of GE on the changes in TBARS levels in brain tissue biomarkers.

Groups	Hippocampus	Cortex	Cerebellum
Normal	4.2 ± 0.11	4.2 ± 0.13	3.5 ± 0.13
AD	7.5 ± 0.09 ^a^	7.9 ± 0.23 ^a^	5.9 ± 0.08 ^a^
AD + GE (2)	4.9 ± 0.05 ^b^	4.7 ± 0.14 ^b^	4.3 ± 0.11 ^b^
AD + GE (4)	4.3 ± 0.02 ^b^	4.3 ± 0.15 ^b^	3.7 ± 0.09 ^b^
AD + DP (1)	4.2 ± 0.03 ^b^	4.2 ± 0.08 ^b^	3.6 ± 0.14 ^b^

Digits in parentheses indicate the dose in mg/kg; the value of TBARS level is expressed as nmol/mg of protein. Data are expressed as the mean ± SD, *n* = 8 mice per group. ^a^ *p* < 0.05 vs. normal group; ^b^ *p* < 0.05 vs. AD group. Abbreviations: AD, Alzheimer’s disease; DP, donepezil; GE, gefitinib.

**Table 3:** Effect of GE on the changes in GSH levels in brain tissue biomarkers.

Groups	Hippocampus	Cortex	Cerebellum
Normal	81.6 ± 1.4	88.1 ± 1.3	74.6 ± 2.1
AD	52.9 ± 0.5 ^a^	58.5 ± 1.6 ^a^	54.2 ± 0.8 ^a^
AD + GE (2)	66.9 ± 1.6 ^b^	70.2 ± 2.1 ^b^	66.9 ± 1.7 ^b^
AD + GE (4)	77.6 ± 1.2 ^b^	83.7 ± 2.2 ^b^	71.7 ± 2.2 ^b^
AD + DP (1)	79.7 ± 1.5 ^b^	86.4 ± 1.3 ^b^	73.1 ± 1.2 ^b^

Digits in parentheses indicate the dose in mg/kg; the value of GSH level is expressed as μmol/mg of protein. Data are expressed as the mean ± SD, *n* = 8 mice per group. ^a^ *p* < 0.05 vs. normal group; ^b^ *p* < 0.05 vs. AD group. Abbreviations: AD, Alzheimer’s disease; DP, donepezil; GE, gefitinib.

**Table 4:** Behavioral assessment patterns of days vs. quadrants.

Days	Quadrants			
Day 1	Q_1_	Q_2_	Q_3_	Q_4_
Day 2	Q_2_	Q_3_	Q_4_	Q_1_
Day 3	Q_3_	Q_4_	Q_1_	Q_2_
Day 4	Q_4_	Q_1_	Q_2_	Q_3_

## Data Availability

The data presented in this study are available on request from the corresponding author.

## Sample Availability

Samples of the compounds are available from the authors.
